# Energy Expenditure Homeostasis Requires ErbB4, an Obesity Risk Gene, in the Paraventricular Nucleus

**DOI:** 10.1523/ENEURO.0139-23.2023

**Published:** 2023-09-25

**Authors:** Ivan Santiago-Marrero, Fang Liu, Hongsheng Wang, Emily P. Arzola, Wen-Cheng Xiong, Lin Mei

**Affiliations:** 1 Department of Neurosciences, School of Medicine, Case Western Reserve University, Cleveland, OH 44106; 2 Louis Stokes Cleveland Veterans Affairs Medical Center, Cleveland, OH 44106; 3 Department of Neuroscience and Regenerative Medicine, Medical College of Georgia, Augusta University, Augusta, GA 30912; 4 Chinese Institutes for Medical Research, Beijing 100005, China; 5 Capital Medical University, Beijing 100054, China

**Keywords:** ErbB4, metabolism, PVH

## Abstract

Obesity affects more than a third adult population in the United States; the prevalence is even higher in patients with major depression disorders. GWAS studies identify the receptor tyrosine kinase ErbB4 as a risk gene for obesity and for major depression disorders. We found that ErbB4 was enriched in the paraventricular nucleus of the hypothalamus (PVH). To investigate its role in metabolism, we deleted ErbB4 by injecting a Cre-expressing virus into the PVH of ErbB4-floxed male mice and found that PVH ErbB4 deletion increased weight gain without altering food intake. ErbB4 PVH deletion also reduced nighttime activity and decreased intrascapular brown adipose tissue (iBAT) thermogenesis. Analysis of covariance (ANCOVA) revealed that ErbB4 PVH deletion reduced O_2_ consumption, CO_2_ production and heat generation in a manner independent of body weight. Immunostaining experiments show that ErbB4+ neurons in the PVH were positive for oxytocin (OXT); ErbB4 PVH deletion reduces serum levels of OXT. We characterized mice where ErbB4 was specifically mutated in OXT+ neurons and found reduction in energy expenditure, phenotypes similar to PVH ErbB4 deletion. Taken together, our data indicate that ErbB4 in the PVH regulates metabolism likely through regulation of OXT expressing neurons, reveal a novel function of ErbB4 and provide insight into pathophysiological mechanisms of depression-associated obesity.

## Significance Statement

Obesity is associated with depression; however, underlying mechanisms are not well understood. Studies have shown that the receptor tyrosine kinase ErbB4 is a risk gene for both obesity and major depression disorder. We found that ErbB4 was expressed in a brain region known for metabolic control. When ErbB4 was deleted in the region, mice became obese. Further studies indicate that ErbB4 is necessary for energy metabolism and for the expression of oxytocin (OXT), a hormone known to regulate metabolism. Energy metabolism was also compromised in mice lacking ErbB4 in oxytocin-expressing neurons. These results uncover a novel mechanism to control metabolism and provide insight into pathophysiological mechanisms of depression-associated obesity.

## Introduction

Obesity affects 42% of adults in the United States (Hales et al., 2020); the prevalence is higher in patients with major depression disorders (Pratt and Brody, 2014). Various brain regions have been implicated in control of body weight, and energy expenditure including the paraventricular nucleus of the hypothalamus (PVH). For example, lesion of the PVH results in obesity in rats (Weingarten et al., 1985). Bilateral ablation of PVH cells that are positive for single-minded 1 (SIM1), a transcription factor that is PVH specific, increased body weight, and reduced energy expenditure (Xi et al., 2012). These phenotypes were also observed in mice with SIM1 haploinsufficiency (Michaud et al., 1998, 2001; Holder et al., 2000). These results demonstrate the role of the PVH in regulating weight gain and serves as an important brain region for the genetic predisposition to obesity.

The PVH consists of various types of cells that express peptidergic hormones including oxytocin (OXT), vasopressin (AVP), among others, to regulate food intake, stress, and energy expenditure (Michaud et al., 1998). Mice with OXT receptor deficiency increase body weight and fat mass (Takayanagi et al., 2008). Conversely, intraventricular administration of OXT decreases food intake in rats and nonhuman primates (Arletti et al., 1990; Olson et al., 1991; Blevins et al., 2016). In accord, ablating OXT-positive cells in mice increases body weight in mice fed with high-fat diets (Wu et al., 2012). Taken together, these results suggest a role of OXT in regulating body weight and fat metabolism.

Recent studies indicate an increasing role of genetics in the development of obesity. Compared with the general population, the risk of obesity is higher in subjects whose parents or monozygotic siblings suffer from metabolic disorders (Stunkard et al., 1990). *de novo* mutations have been identified in the gene *Obese* (that encodes leptin), *Sim1* and *Mc4r* (Montague et al., 1997; Vaisse et al., 1998; Holder et al., 2000). Studies of these risk genes in animal models have revealed insight into pathophysiological mechanisms of obesity.

ErbB4 is a receptor tyrosine kinase of the ErbB family that is activated by growth factors of the neuregulin (NRG) family (Mei and Xiong, 2008; Mei and Nave, 2014). ErbB4 is expressed in the brain as well as in peripheral tissues including the liver, heart, brown and white fats. In the cortex (CTX), hippocampus, and amygdala, ErbB4 is expressed in GABAergic interneurons (INs) and regulates GABAergic transmission and has been implicated in various brain functions (Flames et al., 2004; Y.J. Chen et al., 2010; Neddens and Buonanno, 2010; Y.H. Chen et al., 2017; Tan et al., 2018; Robinson et al., 2022). Interestingly, a GWAS meta-analysis of 339 224 individuals linked ErbB4 in one of the BMI-associated 97 loci; SNP analysis identified ErbB4 as top six risk genes per *p*-values (Locke et al., 2015). Variants in ErbB4 have been associated with BMI in an African American population and associated with severe obesity in a Han Chinese population (Salinas et al., 2016; Chiang et al., 2019). These results suggest that ErbB4 may be a risk gene for obesity. Interestingly, ErbB4 is also a risk gene for major depression disorder (Howard et al., 2019). Recent studies provided evidence that NRG4 produced in fat tissues activates ErbB4 in liver cells to regulate metabolism (G.X. Wang et al., 2014; P. Zhang et al., 2018). Moreover, a recent study found that intracerebroventricular NRG4 administration, decreases mouse body weight, reduces food intake an effect blocked by OXT neuron ablation (Y. Zhang et al., 2023).

Our earlier study indicates that ErbB4 is expressed subcortical regions of the brain including the PVH (Bean et al., 2014), which prompted us to investigate the role of ErbB4 in regulating metabolism. We explored the consequence of regionally ablating ErbB4 using a viral strategy on weight gain and energy expenditure in mice fed a standard chow. Mice were subjected to metabolic chamber recordings to assess deficits in food intake, energy expenditure and locomotor activity. Moreover, we determined the effects of PVH ErbB4 ablation BAT thermogenesis. We determined in which types of cells that ErbB4 was expressed and deleted ErbB4 specifically in OXT-expressing cells. Our results reveal a previously unappreciated role of ErbB4 in the brain in regulating metabolism by through maintaining BAT thermogenesis likely by controlling OXT neurons.

## Materials and Methods

### Animals

Experiment procedures and protocols with mice were approved by the Institutional Animal Care and Use Committee. *ErbB4-T2A-CRE-ERT2-D* (Madisen et al., 2010; JAX #012360) and *Rosa-tdTomato* (Ai14; Madisen et al., 2010; JAX #007908) mice were obtained from Jackson Laboratory. *ErbB4^f/f^* mice were described previously (Tamamaki et al., 2003; García-Rivello et al., 2005; H. Wang et al., 2018). *Oxytocin-IRES-Cre* mice were obtained from Jackson laboratory (Wu et al., 2012), JAX #024234. Mice were housed no more than five per cage at 23°C with a 12/12 h light/dark cycle with food and water available *ad libitum*. Mice were fed with P3000 chow (Prolab RMH 3000).

### Virus injection

Stereotaxic injections were performed as described previously (H. Wang et al., 2021). AAV5-CMV-GFP and AAV5-CMV-Cre-GFP viruses were prepared by Gene Therapy Center Vector Core, the University of North Carolina at Chapel Hill (titer of 10^12^ unit/ml). Glass pipettes (Drummond catalog #5000-1001-X10) were pulled with a micropipette puller (P1000, Sutter). Pipette tips were heat polished (Micro Forge MF-830, Narishige), with their diameters to be 30–50 μm. Two-month-old male *ErbB4^f/f^* mice were anesthetized with ketamine and xylazine (100 and 10 mg/kg, respectively, i.p.) and head-fixed unto a stereotaxic device (David Kopf Instruments). The skull was exposed via a small incision and two small holes were drilled into the skull (bregma: AP: −0.70 mm, ML: ±0.20 mm). Glass pipettes were attached to a 2.5-μl Hamilton syringe and inserted into the brain (DV: −4.75); viruses were injected bilaterally (200 nl, each side) at 50 nl/min by an Ultra Micro Pump (World Precision Instruments). Syringes were remained in the brain for 10 min after injection before being removed. Surgical procedures were performed under antiseptic conditions. Injection sites were verified by postexperimental immunohistochemical analysis (see below).

### Metabolic characterization

Male mice were subjected to metabolic analysis by the Comprehensive Lab Animal Monitoring System (CLAMS, Columbus Instruments), or the Promethion metabolic cage (Sable Systems) as described previously (Li et al., 2019). Indirect calorimetry, locomotor activity and food intake were monitored noninvasively. Fat and lean mass were measured by Nuclear Magnetic Resonance (minispec LF90 TD-NMR, Bruker).

### Brown adipose tissue (BAT) temperature measurement

BAT temperature was measured as described previously(Edwards et al., 2021) with modifications. The interscapular region of anesthetized mice was shaved and cleaned by antiseptic procedures. A small incision was made and implanted with an electronic temperature transponder (IPTT-300, Bio Medic Data Systems). The transponder was secured to BAT, and the scapular incision was closed by suture. One week after surgery, BAT temperature was measured in free-moving mice using a handheld reader (DAS-8007B-IUS, Bio Medic Data Systems).

### Immunohistochemistry

Immunohistochemical analysis was performed as previously described with modifications (H. Wang et al., 2018). Mice were deeply anesthetized with isoflurane in a bell jar with two chambers and intracardiacally perfused with 50 ml of 0.1 m PBS (pH 7.4) followed by freshly prepared 4% paraformaldehyde in PBS. Brains were removed and postfixed with 4% paraformaldehyde overnight. Brains were incubated in 15% sucrose in PBS 12 h at 4°C and 30% sucrose in PBS for additional 24 h. Coronal hypothalamus sections (20–40 μm in thickness) were sliced in a VT-1000S vibratome (Leica) and placed on a 12-well plated and washed three times in 0.05 M. Tris-buffered saline (TBS, pH 7.2), each for 10 min on a shaker. Sections were then permeabilized with PBS with 0.5% Triton X-100 (Fisher catalog #AAA16046AE) for 15 min at room temperature. Sections were then incubated with the blocking buffer containing PBS, 0.5% Triton X-100 and 10% donkey serum (Millipore, S-30) for 30 min on a shaker and with primary antibodies in the blocking buffer for 24 h at 4°C. After washing three times with TBS, each for 10 min on a shaker, sections were incubated with Alexa488-conjugated secondary antibodies for visualization. Sections were incubated with secondary antibodies at room temperature for 1–2 h and washed three times with TBS each for 10 min and washed. Some sections were incubated with 4’,6’-diamidino-2-phenylindole (DAPI; Sigma catalog #28718-90-3) for 5 min in TBS. Sections were mounted on a slide with AquaMount (Lerner Laboratories catalog #13800) with coverslips for imaging using a Zeiss LSM7800 confocal laser scanning microscope (with a 20× objective). Z-stack confocal images were collapsed with Zeiss LSM software.

Antibodies used were as follows: mouse anti-NeuN (1:1000, EMD Millipore, catalog #MAB377), rabbit anti-oxytocin (1:1000, Peninsula Laboratories International, catalog #T-4084), rabbit anti-vasopressin (1:1000, EMD Millipore, catalog #PC234L), anti-rabbit Tyrosine Hydroxylase (1:1000, EMD Millipore, catalog #AB152). Alexa488-conjugated anti-mouse and anti-rabbit (1:500, Jackson ImmunoResearch catalog #715-545-150) and anti-mouse (1:500, Jackson ImmunoResearch catalog #711-545-152).

### Western blotting analysis

Brain tissues were dissected and lysed for 15 min in ice-cold modified RIPA buffer (50 mm Tris-HCl, pH 7.4, 150 mm NaCl, 2 mm EDTA, 1 mm phenylmethylsulfonyl fluoride (PMSF), 50 mm sodium fluoride, 1 mm sodium vanadate, 1% sodium deoxycholate, 1% SDS, and 1× protease inhibitors cocktail (Roche catalog #04693159001). Samples were cleared by centrifugation at 12,000 × *g* for 20 min at 4°C; the supernatants were resolved on 8% SDS-PAGE and transferred to nitrocellulose membranes (Bio-Rad catalog #1620112) using a wet-transfer system at 100 V for 2 h. Membranes were then incubated with 5% skim milk in TBS containing 1% Triton X-20 (TBST). Membranes were washed three times with TBST, each for 10 min and incubated overnight with primary antibodies for ErbB4 (1:2000, #0618, generously provided by Cary Lai) and GAPDH (1:10,000, Cell Signaling, catalog #97166S) in 5% bovine serum albumin in TBST. Membranes were washed three times with TBST, each for 10 min and incubated with anti-rabbit or anti-mouse horse radish peroxidase (HRP)-conjugated secondary antibodies (1:1000, Invitrogen, catalog #A16029 and #31430, for rabbit and mouse, respectively) for 1 h at room temperature. After washing three times with TBST (each for 10 min), immunoreactive bands on the membranes were visualized using enhanced chemiluminescence substrate kit (ThermoFisher catalog #32106). Images were captured using an Odyssey Fc infrared imaging system (LI-COR) and analyzed using ImageStudio software provided by the manufacturer.

### ELISA

Serum levels of OXT was measured as described previously (Lawson et al., 2012). Sera was incubated with equal volumes of 0.1% trifluoracetic acid (TFA, Sigma catalog #302031) in water. The reaction was centrifuged at 17.000 × *g* for 15 min at 4°C) and the supernatant subjected to chromatography on a C18-Sep Pak column (Waters catalog #WAT054955) that was preconditioned with 0.1% TFA in water. Columns were eluted using 60% acetonitrile 0.1% TFA in water. Eluates were stored frozen and later thawed and assayed with respective ELISA kits (Enzo catalog #ADI-900-153A-000 for OXT, sensitivity = 15–1000 pg/ml, mean intra-assay variability = 12.0) according to manufacturers’ instructions.

### Statistics

**Figure 1.**
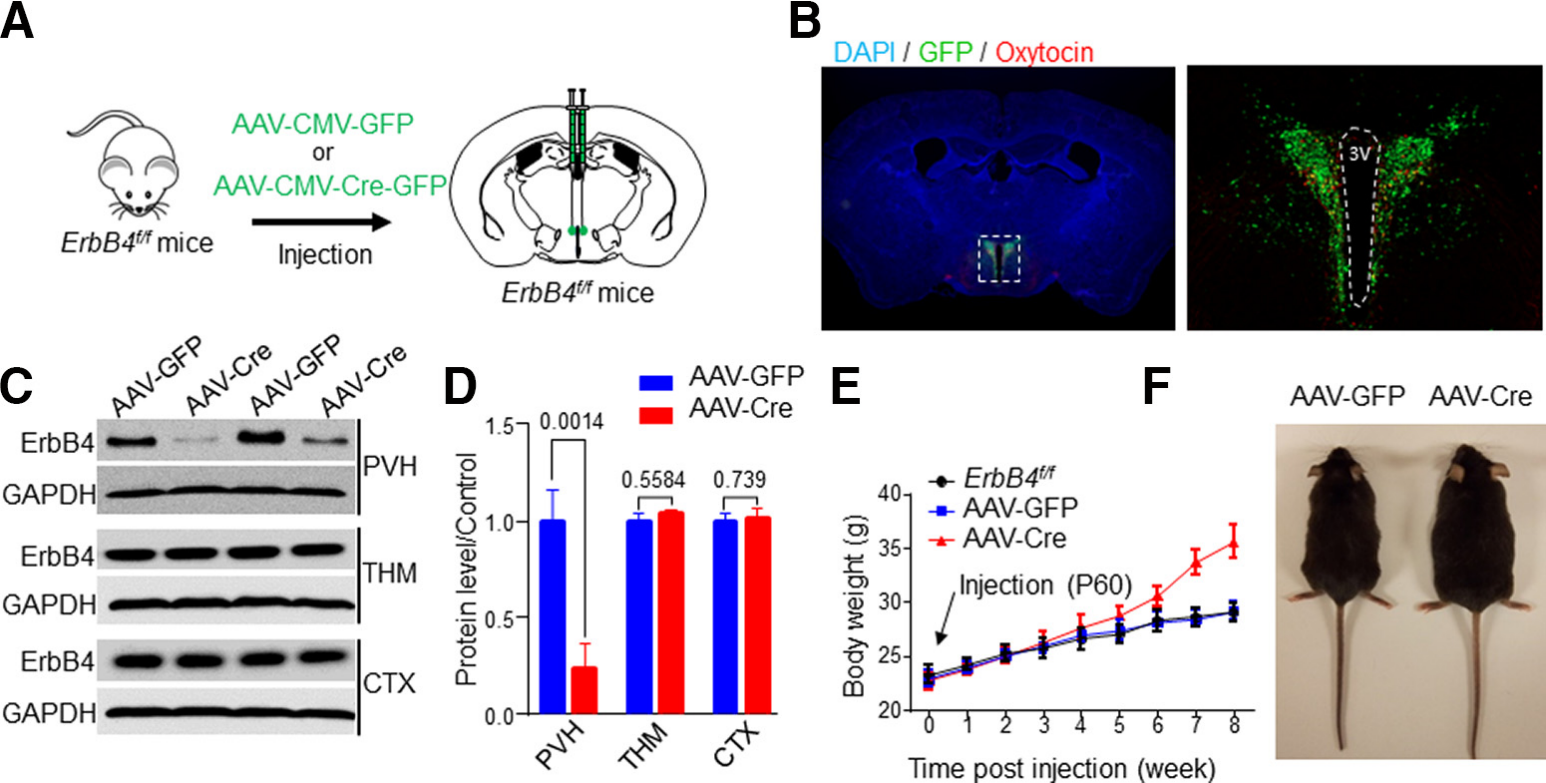
PVH ErbB4 deletion increased body weight. ***A***, Diagram of viral strategy to delete ErbB4 in the PVH. ***B***, Viral GFP expression in the PVH. Mice were injected with AAV-GFP. PVH sections were visualized for GFP. ***C***, Specific reduction of ErbB4 in PVH, not the thalamus (THM) or the cortex (CTX). ***D***, Quantification of data in ***C***. ***E***, ***F***, Increased body weight in AAV-Cre mice, compared with AAV-GFP mice or mice which received no injection (*ErbB4^f/f^*). Data are presented as mean ± SEM; *p* > 0.05, not significant, *p < 0.5, **p < 0.01.

**Figure 2.**
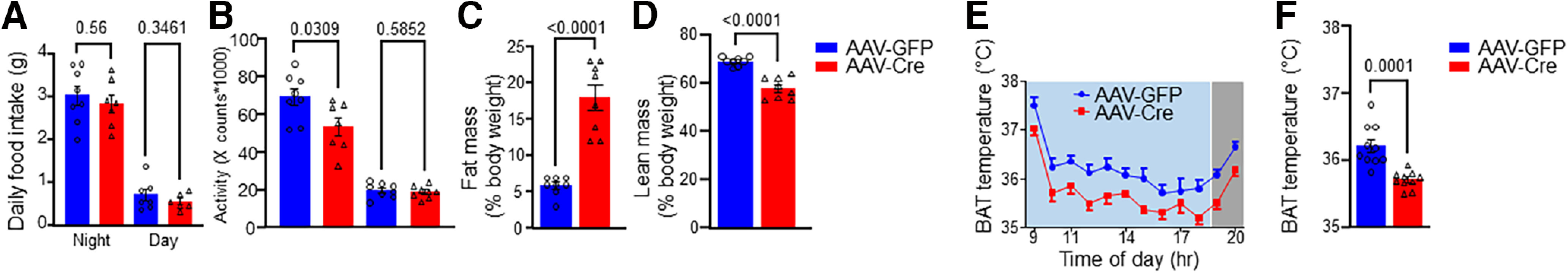
Reduced fat mass and intrascapular BAT temperature in AAV-Cre mice. Mice were injected with either AAV-GFP (control) and AAV-Cre virus; one month after injection, they were subjected to CLAMS, to measure food intake and activity. ***A***, Similar food intake between AAV-GFP and AAV-Cre mice was observed. ***B***, Reduced nighttime locomotor activity in mice with PVH ErbB4 deletion but not daytime locomotor activity. ***C***, ***D***, Mice with PVH ErbB4 deletion showed increased fat mass, and reduced lean mass. ***E***, ***F***, Reduction in intrascapular BAT thermogenesis after PVH ErbB4 deletion. Light blue square indicates day and gray indicates night. Data are presented as mean ± SEM; *p* > 0.05, not significant, *p < 0.5, **p < 0.01, ***p < 0.001.

**Figure 3.**
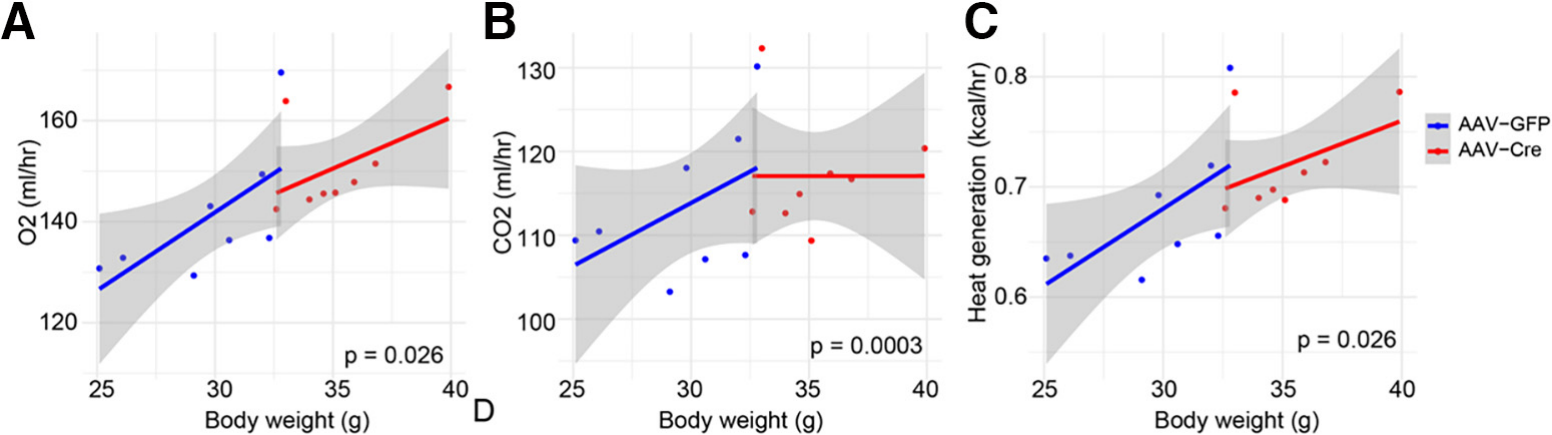
PVH ErbB4 deletion reduced mouse energy expenditure. ***A***, Reduced O_2_ consumption in AAV-Cre mice, compared with AAV-GFP mice. ***B***, Reduced CO_2_ production in AAV-Cre mice, compared with AAV-GFP mice. ***C***, Reduced heat generation in AAV-Cre mice. Significance levels in panels ***A–C*** illustrates effects of ErbB4 deletion from the ANCOVA analysis. Shaded areas indicate confidence intervals.

**Figure 4.**
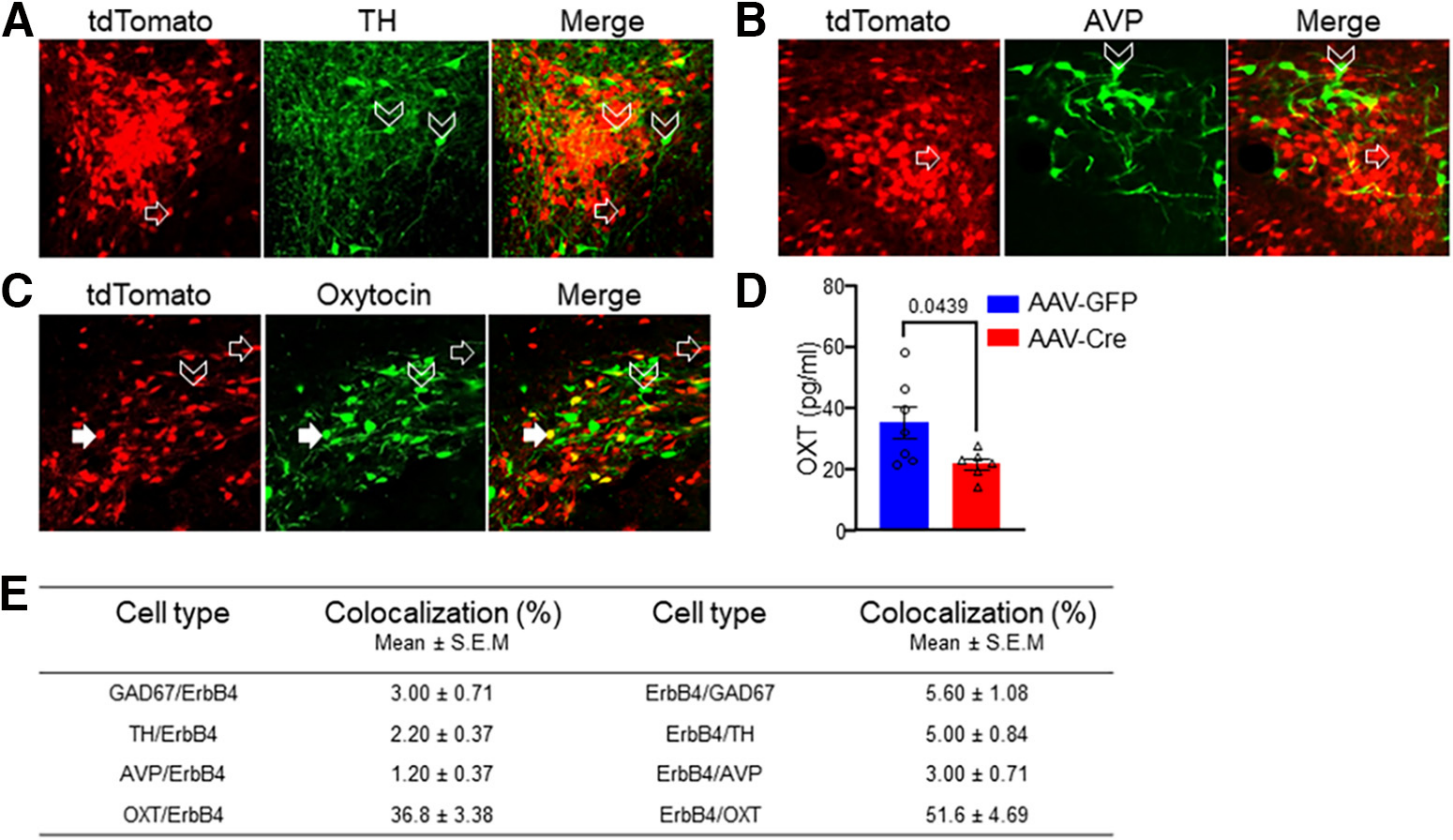
ErbB4+ cells in the PVH were positive for OXT. ***A–C***, ErbB4+ cells in the PVH were not positive for TH (***A***) or AVP (***B***), but for OXT (***C***). Brain slices of *ErbB4-tdTomato* mice were stained with respective antibodies that were visualized by Alexa488-conjugated secondary antibody (green). ***D***, Reduced serum levels of OXT in AAV-Cre mice, compared with AAV-GFP. ***E***, Quantification of immunostaining data. Data are presented as mean ± SEM; *p < 0.05.

**Figure 5.**
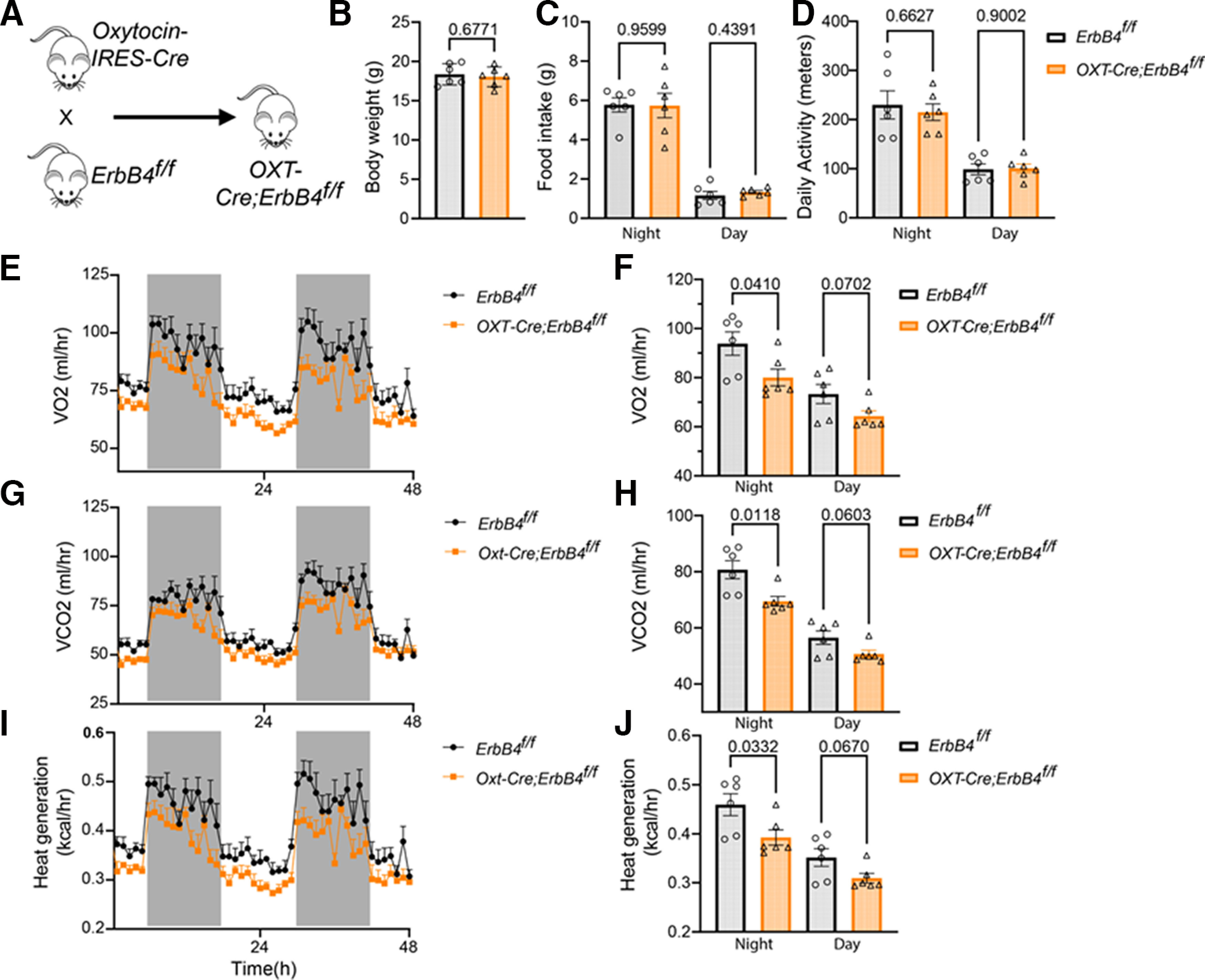
Specific deletion of ErbB4 in OXT+ cells reduced energy expenditure. OXT-Cre mice were crossed with *ErbB4^f/f^* to generate *OXT-Cre;ErbB4^f/f^* mice. They were subjected to metabolic measurements as described in methods, with *ErbB4^f/f^* as control. ***A***, Breeding diagram. ***B***, *ErbB4^f/f^* and *OXT-Cre;ErbB4^f/f^* mice used for recording did not differ in body weight. ***C,*** Similar food intake of OXT-Cre;ErbB4^f/f^ mice during the nighttime and daytime, compared with their *ErbB4^f/f^* littermates. ***D***, Similar levels of locomotor activity between *OXT-Cre;ErbB4^f/f^* and *ErbB4^f/f^* mice during the nighttime and daytime. ***E***, ***F***, Reduced O_2_ consumption between *OXT-Cre;ErbB4^f/f^* mice, compared with *ErbB4^f/f^* mice during the nighttime. **G-*H*,** CO_2_ production was reduced in *OXT-Cre;ErbB4^f/f^* mice, compared with *ErbB4^f/f^* mice during the nighttime. ***I***, ***J***, Reduced heat generation during the nighttime in *OXT-Cre;ErbB4^f/f^* mice, compared with *ErbB4^f/f^* mice. Data are presented as mean ± SEM; *p* > 0.05, not significant, *p < 0.5, **p < 0.01.

Data for Figures 1, 2, 4, and 5 were plotted and analyzed using GraphPad Prism version 9.4 for statistical significance. Data of two groups were analyzed using Student’s *t* test. Data are presented as mean ± SEM. Data for Figure 3 were plotted and analyzed using R Statistical Software (v4.2.2; R Core Team 2022). Analysis of covariance (ANCOVA) using the *stats* (v4.2.2 R Core Team, 3033) and *car* (v3.1.2; Fox and Weisberg, 2019) packages. Data for Figures 4, 5 were plotted and analyzed using GraphPad Prism version 9.4 for statistical significance. Exact *p*-values are listed in Table 1 and on figure panels. *p*-values < 0.05 were considered significant.

**Table 1:** Summary of statistical analysis

Figure #	Panel	Comparison	Data structure: normality	Type of test	95% confidence interval	*p*-value
Fig. 1	*D*	AAV-GFP vs AAV-Cre	Normal distribution Shapiro–Wilk test PVH: *W* = 0.9004, *p*-value = 0.2915 THM: *W* = 0.9265, *p* = 0.4847 CTX: *W* = 0.9329, *p* = 0.5433	Unpaired Student’s *t* test	PVH: −1.034 to −0.4045 THM: −0.2085 to 0.3499 CTX: −0.1340 to 0.2498	PVH = 0.0014 THM = 0.5584 CTX = 0.739
Fig. 2	*A*	Night AAV-GFP vs Night AAV-Cre, Day AAV-GFP vs Day AAV-Cre	Normal distribution Shapiro–Wilk test Night: *W* = 0.9449, *p*-value = 0.4481 Day: *W* = 0.9096, *p* = 0.1813	Unpaired Student’s *t* test	Night :- −0.8609 to 0.4879 Day: −0.5116 to 0.1954	Night = 0.56 Day = 0.3461
Fig 2	*B*	Night AAV-GFP vs Night AAV-Cre, Day AAV-GFP vs Day AAV-Cre	Normal distribution Shapiro–Wilk test Night: *W* = 0.9404, *p*-value = 0.3872 Day: *W* = 0.9747, *p* = 0.9080	Unpaired Student’s *t* test	Night: −29.94 to −1.701 Day: −4.874 to 2.859	Night = 0.0309 Day = 0.5852

Fig. 2	*C*	AAV-GFP vs AAV-Cre	Normal distribution Shapiro–Wilk test *W* = 0.9415, *p*-value = 0.3679	Unpaired Student’s *t* test	8.313 to 15.94	<0.0001
Fig. 2	*D*	AAV-GFP vs AAV-Cre	Normal distribution Shapiro–Wilk test *W* = 0.9632, *p*-value = 0.7206	Unpaired Student’s *t* test	−14.76 to −7.490	<0.0001
Fig. 2	*F*	AAV-GFP vs AAV-Cre	Normal distribution Shapiro–Wilk test *W* = 0.9374, *p*-value = 0.2139	Unpaired Student’s *t* test	−0.7195 to −0.2805	0.0001
Fig. 3	*A*	AAV-GFP vs AAV-Cre	Not normally distributed	ANCOVA	3.65 to 57.90	0.0261
Fig. 3	*B*	AAV-GFP vs AAV-Cre	Not normally distributed	ANCOVA	22.02 to 74.35	0.0003
Fig. 3	*C*	AAV-GFP vs AAV-Cre	Not normally distributed	ANCOVA	0.028 to 0.3042	0.018
Fig. 4	*D*	AAV-GFP vs AAV-Cre	Normal Distribution Shapiro–Wilks test *W* = 0.9478, *p*-value = 0.5658	Unpaired Student’s *t* test	−26.22 to −0.4339	0.0439
Fig. 5	*B*	*ErbB4^f^*^/^*^f^* vs *OXT-Cre*;*ErbB4^f^*^/^*^f^*	Normal distribution Shapiro–Wilk test *W* = 0.9253, *p*-value = 0.3325	Unpaired Student’s *t* test	−2.006 to 0.7549	0.6771

Fig. 5	*C*	*ErbB4^f^*^/^*^f^* vs *OXT-Cre*;*ErbB4^f^*^/^*^f^*	Normal distribution Shapiro–Wilk test Night: *W* = 0.9412 *p*-value = 0.5136 Day: *W* = 0.9473, *p*-value = 0.5975	Unpaired Student’s *t* test	Night: −1.637 to 1.563 Day: −0.3115 to 0.6644	Night = 0.9599 Day = 0.4391
Fig. 5	*D*	Night *ErbB4^f^*^/^*^f^* vs Night *OXT-Cre;ErbB4^f^*^/^*^f^* Day ErbB4*^f^*^/^*^f^* vs Day *OXT-Cre;ErbB4^f^*^/^*^f^*	Normal distribution Shapiro–Wilk test Night: *W* = 0.9412, *p*-value = 0.5136 Day: *W* = 0.9244, *p* = 0.3248	Unpaired Student’s *t* test	Night: −89.10 to 59.19 Day: −29.74 to 33.39	Night = 0.6627 Day = 0.9002
Fig. 5	*F*	Night ErbB4*^f^*^/^*^f^* vs Night *OXT-Cre;ErbB4^f^*^/^*^f^*, Day *ErbB4^f^*^/^*^f^* vs Day *OXT-Cre;ErbB4^f^*^/^*^f^*	Normal distribution Shapiro–Wilk test Night: *W* = 0.9475, *p*-value = 0.6010 Day: *W* = 0.9404, *p* = 0.5034	Unpaired Student’s *t* test	Night: −26.89 to −0.6874 Day: −18.97 to 0.8983	Night = 0.0410 Day = 0.0702
Fig. 5	*H*	Night *ErbB4^f^*^/^*^f^* vs Night *OXT-Cre;ErbB4^f^*^/^*^f^* Day *ErbB4^f^*^/^*^f^* vs Day *OXT-Cre;ErbB4^f^*^/^*^f^ ^f^*	Normal distribution Shapiro–Wilk test Night: *W* = 0.9081, *p*-value = 0.2015 Day: *W* = 0.9206, *p*-value = 0.2906	Unpaired Student’s *t* test	Night: −19.39 to −3.092 Day: −11.98 to 0.3069	Night = 0.0118 Day = 0.0603

Fig. 5	*J*	Night *ErbB4^f^*^/^*^f^* vs Night *OXT-Cre;ErbB4^f^*^/^*^f^*, Day *ErbB4^f^*^/^*^f^* vs Day *OXT-Cre;ErbB4^f^*^/^*^f^*	Normal distribution Shapiro–Wilk test Night: *W* = 0.9499, *p*-value = 0.6349 Day: *W* = 0.9325, *p* = 0.4077	Unpaired Student’s *t* test	Night: −0.1271 to 0.006495 Day: −0.08767 to 0.003547	Night = 0.0332 Day = 0.0670

## Results

### PVH-specific deletion of ErbB4 increases body weight

To determine whether ErbB4 in the PVH regulates metabolism, we sought to explore the consequences of ErbB4 knock-down in the region. ErbB4f/f mice [two months old (mo)] were bilaterally injected into the PVH with AAV-CMV-Cre-GFP, an adeno-associated virus (AAV) expressing Cre-GFP under the control of the CMV promoter whereas control mice were injected with AAV-CMV-GFP (Fig. 1*A*). These mice were referred to as AAV-Cre and AAV-GFP, respectively. Postmortem analysis revealed GFP expressed by injected virus in bilateral PVH regions of AAV-GFP mice (Fig. 1*B*). One month after injection, brain samples were subjected to western blotting to determine whether ErbB4 was deleted in the PVH by viral Cre. As shown in Figure 1*C*, ErbB4 levels were reduced in the PVH of AAV-Cre mice, compared with samples of AAV-GFP mice. The reduction appeared to be region-specific because ErbB4 levels in the thalamus (THM) and cortex (CTX) were similar between AAV-GFP and AAV-Cre mice (for PVH, *n* = 4, *t*_(6)_ = 5.59 *p* = 0.0014, unpaired *t* test; for THM, *n* = 4, *t*_(6)_ = 0.62, *p* = 0.5584, unpaired *t* test; for CTX, *n* = 4, *t*_(6)_ = 0.739, *p* = 0.4881, unpaired *t* test; Fig. 1*D*). These results indicate that ErbB4 was specifically reduced in the PVH by the viral targeting strategy. Mice were fed with a standard chow and monitored for body weight. The body weights of ErbB4^f/f^ mice and AAV-GFP mice remained similar during the eight weeks of experiments, suggesting that AAV-CMV-GFP injection had little effect on the body weight. In contrast, the body weight of AAV-Cre mice showed a trend of increase at six weeks after viral injection. Seven weeks after AAV injection, AAV-Cre mice showed a significant increase in body weight when compared with AAV-GFP or *ErbB4^f/f^* mice (Fig. 1*E*,*F*). At eight weeks after injection, the body weight of AAV-Cre mice increased by 20% of that of AAV-GFP mice (Fig. 1*E*). These results identified a role of ErbB4 in the PVH in regulating the body weight.

Physiologic mechanisms of the body weight increase could be complex. To this end, first, we monitored food intake and locomotor activity of free-moving mice using the CLAMS (Fig. 2). Food consumption is more during nighttime over daytime, in nocturnal behavior of rodents (Garfield et al., 2015). In agreement, food intake was more during nighttime for both AAV-GFP and AAV-Cre. However, the two groups showed no difference in the amount of chow consumption (for nighttime, AAV-GFP, 3.04 ± 0.23, AAV-Cre, 2.85 ± 0.21, *t*_(13)_ = 0.60, *p* = 0.56, unpaired *t* test; for daytime, AAV-GFP, 0.72 ± 0.13, AAV-Cre 0.56 ± 0.08, *t*_(11)_ = 0.98, *p* = 0.35 unpaired *t* test; Fig. 2*A*) as measured by the CLAMS metabolic cage system, suggesting that PVH ErbB4 deletion-induced body weight increase may not be because of increased food intake. Next, we compared the locomotor activity between AAV-GFP and AAV-Cre mice. As shown in Figure 2*B*, both groups of mice were more active during the nighttime than daytime. Locomotor activity was similar during the daytime between the two groups (AAV-GFP, 19.86 ± 1.38, AAV-Cre, 18.86 ± 1.17, *t*_(14)_ = 0.56 *p* = 0.59, unpaired *t* test; Fig. 2*B*). However, a 25% decrease in locomotor activity was observed during the nighttime in AAV-Cre mice, compared with AAV-GFP mice (AAV-GFP, 69.3 ± 4.33, AAV-Cre 53.5 ± 4.93, *t*_(13)_ = 2.42, *p* = 0.03, unpaired *t* test; Fig. 2*B*). These results could suggest that body weight increase by PVH ErbB4 deletion may result from a reduction in nighttime activity.

### Increased fat mass by PVH ErbB4 deletion

To further understand cellular mechanisms, we analyzed composition of the whole body, as described in Methods. Intriguingly, fat mass accounted 18 ± 1.71% and 5.9 ± 1.71% in AAV-Cre and AAV-GFP mice (*n* = 8, *t*_(14)_ = 6.82 *p* < 0.0001, unpaired *t* test; Fig. 2*C*), respectively, indicating an 220% increase in body fat mass by PVH ErbB4 mutation. Concomitantly, the lean mass in AAV-Cre mice was less than AAV-GFP mice (57.9 ± 1.58% and 69.0 ± 0.63%, respectively, *n* = 8, *t*_(14)_ = 6.56, *p* < 0.0001, unpaired *t* test; Fig. 2*D*), indicating a reduction of lean mass by PVH ErbB4 deletion.

Earlier studies indicated that fat mass increase by genetic ablation or physical lesion of the PVH results from an increase in food intake (Weingarten et al., 1985; Holder et al., 2000; Michaud et al., 2001; Balthasar et al., 2005). However, because food intake was not increased in AAV-Cre mice, we posited that increased fat mass may be caused by reduced thermogenesis BAT. Therefore, we measure the temperature of the scapular BAT in free-moving mice. Because the nighttime activity of AAV-Cre mice was increased, which is likely to increase the BAT temperature, we focused on BAT temperature in the daytime. As shown in Figure 2*E*,*F*, daytime temperature of scapular BAT was consistently lower in AAV-Cre mice, compared with AAV-GFP mice (AAV-GFP, 36.2 ± 0.06°C, AAV-Cre, 35.7 ± 0.06°C, *n* = 10, *t*_(238)_ = 5.84 *p* < 0.0001, unpaired *t* test; Fig. 2*E*,*F*). These results support a model where PVH ErbB4 deletion increases fat mass by reducing BAT thermogenesis.

### Reduced energy expenditure in AAV-Cre mice

To investigate mechanisms of fat mass increase, we subjected free-moving mice to indirect calorimetry by CLAMS analysis and measured oxygen (O_2_) consumption, carbon dioxide (CO_2_) production and heat generation, parameters that are well-established indicators of energy expenditure (Michaud et al., 2001; Wu et al., 2012) Because AAV-Cre mice weighted more than AAV-GFP mice (AAV-GFP, *n* = 8, for AAV-Cre *n* = 8, *p* < 0.0009, *t*_(13)_ = 4.22; Fig. 1*E*), parameters were subjected to analysis of covariance (ANCOVA) to determine effects of ErbB4 deletion on energy expenditure as described previously (Müller et al., 2021; Tschöp et al., 2011). ErbB4 PVH deletion reduced O_2_ consumption, CO_2_ production and heat generation in a manner independent of body weight (AAV-GFP, *n* = 8, for AAV-Cre *n* = 8, ANCOVA, *p* = 0.026 for Fig. 3*A*, *p* = 0.0003 for Fig. 3*B*; and *p* = 0.018 for Fig. 3*C*).

### Serum OXT reduction by PVH ErbB4 deletion

Next, we investigated how ErbB4 in PVH regulates energy expenditure. Earlier results indicate that ErbB4 is expressed in GABAergic interneurons (INs) in the cortex, hippocampus, and amygdala (Flames et al., 2004; Woo et al., 2007; Fazzari et al., 2010; Bean et al., 2014). First, we determined whether PVH ErbB4+ neurons were GABAergic. *ErbB4-T2A-CRE-ERT2-D* mice, which express CRE-ERT2 under the promoter of the endogenous ErbB4 gene (Madisen et al., 2010), were crossed with Rosa-LSL-tdTomato mice. Resulting compound mice, referred to as B4-TdTomato mice, express tdTomato specifically in ErbB4+ cells in the brain (Bean et al., 2014). Immunostaining experiments on B4-TdTomato mice show that many (∼37%) of ErbB4+ cells in the PVH were positive for OXT; only 2% and 1% of ErbB4+ cells were positive for TH and AVP, respectively. On the other hand, 52% of OXT+ cells in the PVH co-expressed ErbB4 (Fig. 4*C*,*D*, quantified in *E*). Indeed, the plasma OXT levels in AAV-Cre mice were 38% lower of those in AAV-GFP mice (Fig. 4*D*). Taken together this data suggest that ErbB4 is expressed in the OXT neurons and deletion of ErbB4 in the PVH reduces plasma OXT levels (35.1 ± 5.16 for AAV-GFP, 21.8 ± 1.85 for AAV-Cre, *n* = 7 for AAV-GFP, *n* = 6 for AAV-Cre, *t* = 2.28, *p* = 0.0439, unpaired *t* test; Fig. 4*D*).

### Reduced energy expenditure and food intake by OXT+ cell-specific deletion of ErbB4

The findings that ErbB4 is expressed in OXT+ cells in the PVH raised the possibility that ErbB4 effects on energy expenditure and body weight it is through regulation of OXT neurons of the PVH. To test this hypothesis, we crossed ErbB4^f/f^ mice with *OXT-IRES-Cre* mice (OXT-Cre) that expressed Cre under the promoter of the endogenous OXT gene (Wu et al., 2012), to generate compound *OXT-Cre;ErbB4^f/f^* mice (Fig. 5*A*). *OXT-Cre;ErbB4^f/f^* mice were subjected to energy expenditure measurements (Fig. 5). *OXT-Cre;ErbB4^f/f^* mice subjected to metabolic chamber recordings displayed similar body weight at postnatal day (P)30 that *ErbB4^f/f^* counterparts (18.37 ± 0.55 g for *ErbB4^f/f,^* 18.04 ± 0.5149 g for *OXT-Cre; ErbB4^f/f^*, *n* = 6 for *ErbB4^f/f^*, *n* = 6 for *OXT-Cre;ErbB4^f/f^*, *t*_(10)_ = 0.4289, *p* = 0.6771; Fig. 5*B*). *OXT-Cre;ErbB4^f/f^* showed similar food intake during the nighttime and daytime when compared with *ErbB4^f/f^* mice (nighttime 5.78 ± 0.36 g for *ErbB4^f/f^* and 5.74 ± 0.62 for *OXT-Cre;ErbB4^f/f^*, *n* = 6, *t*_(10)_ = 0.051, *p* = 0.96, unpaired *t* test; daytime 1.163 ± 0.20 g for *ErbB4^f/f^* and 1.339 ± 0.08 for *OXT-Cre;ErbB4^f/f^*, *n* = 6, *t*_(10)_ = 0.81, *p* = 0.44, unpaired *t* test; Fig. 5*C*). Unlike ErbB4 PVH deletion, OXT neuron ErbB4 deletion did not affect nighttime activity (230.0 ± 28.7 for ErbB4^f/f^ and 215.0 ± 16.9 for *OXT-Cre;ErbB4^f/f^*, *n* = 6, *t*_(10)_ = 0.45, *p* = 0.66, unpaired *t* test; Fig. 5*D*). Daytime activity was unchanged after OXT neuron ErbB4 deletion (98.9 ± 11.1 for *ErbB4^f/f^* and 100.7 ± 8.86 for *OXT-Cre;ErbB4^f/f^*, *n* = 6, *t*_(10)_ = 0.13, *p* = 0.90, unpaired *t* test; Fig. 5*D*). *OXT-Cre;ErbB4^f/f^* and *ErbB4^f/f^* displayed circadian fluctuations in O_2_ consumption, CO_2_ production and heat production, suggesting that ErbB4 in OXT+ cells is not required for the circadian rhythm. However, O_2_ consumption during the nighttime was lower in *OXT-Cre;ErbB4^f/f^* mice than that of *ErbB4^f/f^* mice (93.9 ± 4.76 and 80.1 ± 3.46 ml/h at nighttime, respectively, *n* = 6, *t*_(10)_ = 2.36, *p* = 0.04, unpaired *t* test; Fig. 5*E*,*F*). Daytime O_2_ consumption displayed a trending decrease between *ErbB4^f/f^* and *OXT-Cre;ErbB4^f/f^* mice, but did not reach statistical significance. (73.3 ± 3.88 ml/h and 60.3 ± 2.19 at daytime, respectively, *n* = 6, *t*_(10)_ = 2.03, *p* = 0.07, unpaired *t* test; Fig. 5*E*,*F*). CO_2_ production was reduced *OXT-Cre;ErbB4^f/f^* when compared with *ErbB4^f/f^* mice during the nighttime (80.7 ± 3.23 ml/h for *ErbB4^f/f^* and 69.5 ± 1.72 ml/h for *OXT-Cre;ErbB4^f/f^* at nighttime, *n* = 6, *t*_(10)_ = 3.07, *p* = 0.01, unpaired *t* test; Fig. 5*G*,*H*). During the daytime CO_2_ production was also showed a trending decreased between *ErbB4^f/f^* and *OXT-Cre;ErbB4^f/f^* mice but did not reach significance (56.5 ± 2.40 ml/h for *ErbB4^f/f^* and 50.7 ± 1.35 ml/h for *OXT-Cre;ErbB4^f/f^* at daytime, respectively, *n* = 6, *t*_(10)_ = 2.12, *p* = 0.06, unpaired *t* test; Fig. 5*G*,*H*). Nighttime but not daytime heat generation was reduced in *OXT-Cre;ErbB4^f/f^* mice when compared with *ErbB4^f/f^* mice (0.46 ± 0.02 kcal/h and 0.39 ± 0.15 ml/h for *ErbB4^f/f^* and *OXT-Cre;ErbB4^f/f^* mice respectively, *n* = 6, *t*_(10)_ = 2.47, *p* = 0.03, unpaired *t* test; Fig. 5*I*,*J*). During the daytime heat generation *ErbB4^f/f^* mice was 0.35 ± 0.02 kcal/h and 0.31 ± 0.01 kcal/h for *OXT-Cre;ErbB4^f/f^*, respectively (*n* = 6, *t*_(10)_ = 2.06, *p* = 0.07, unpaired *t* test; Fig. 5*I*,*J*). Taken together these data indicate that deletion of ErbB4 in OXT neurons reduces mice energy expenditure independent of food intake and locomotor activity.

## Discussion

This study provides evidence for a role of ErbB4 in the brain to control energy expenditure and body weight. First, ErbB4 deletion in the PVH increased body weight, increased fat mass, and decreased BAT thermogenesis without altering food intake (Figs. 1, 2). Second, to investigate underlying mechanisms, we measured energy expenditure after PVH ErbB4 deletion and showed that it decreased mouse energy expenditure (Fig. 3). Third, we demonstrate that ErbB4 is expressed in OXT neurons of the PVH and that PVH ErbB4 deletion reduces serum levels of OXT (Fig. 4). Finally, we show that deletion of ErbB4 specifically in OXT neurons causes similar energy expenditure phenotypes as PVH ErbB4 deletion.

Earlier studies showed that the NRG-ErbB4 signaling in peripheral tissues is critical to metabolism. ErbB4-null mice are susceptible to diet induced obesity, with increased fasting plasma glucose and insulin and reduced liver function (Zeng et al., 2018). NRG1 injection (intraperitoneal or intracerebroventricular) reduces the body weight and fat, and serum glucose (Snodgrass-Belt et al., 2005; Ennequin et al., 2015a,b; P. Zhang et al., 2018). NRG1 also increases serum leptin and its effect to reduce body weight/fat was not observed in leptin receptor mutant mice (Ennequin et al., 2015b). In an emerging model, NRG4 from fat tissues plays an important role in regulating metabolism by activating ErbB4 in the liver (G.X. Wang et al., 2014). NRG4 null mice showed higher plasma glucose and insulin; its expression is reduced in leptin and leptin-receptor mutant mice (G.X. Wang et al., 2014). On the other hand, transgenic expression of NRG4 decreases plasma glucose and insulin and plasma triglycerides, likely by reducing the expression of lipogenic genes (G.X. Wang et al., 2014). NRG4 mRNA is negatively correlated with body fat and fat liver content in human healthy subjects and diabetic patients (G.X. Wang et al., 2014; Cai et al., 2016).

We showed that PVH ErbB4 deletion reduced energy expenditure. Moreover, similar energy expenditure deficits caused by PVH ErbB4 deletion were observed in *OXT-Cre;ErbB4^f/f^* mice: reduced O_2_, CO_2_ and heat generation. These results suggest that ErbB4 in PVH regulates metabolism. Interestingly, PVH ErbB4 deletion reduced OXT serum level (Fig. 4*D*). Administration of OXT has been shown to suppress feeding (Arletti et al., 1990; Blevins et al., 2015) and decrease fat mass while OXT receptor knock-out increases fat mass and reduces thermogenesis after cold exposure (Takayanagi et al., 2008). Ablation of OXT+ neurons increase body weight and reduces energy expenditure in mice with a high fat diet without affecting food intake (Wu et al., 2012). The association of OXT reduction in the serum with impaired energy expenditure suggest a potential involvement of OXT in PVH ErbB4 regulation of metabolism.

The PVH consists functionally distinct populations of neurons such as neuroendocrine (parvicellular or magnocellular) neurons (Biag et al., 2012; Doslikova et al., 2019; Simmons and Swanson, 2009). Parvicellular neurons release neuropeptides such as CRH and TRH into the hypophysial portal system whereas magnocellular neurons deliver OXT and AVP to the posterior pituitary lobe for release into the blood. In addition to neuroendocrine neurons, the PVH also contains descending and preautonomic neurons that are positive for CRH, cocaine-related and amphetamine-related transcript (CART), or OXT/nitric oxide (NOS); these glutamatergic neurons project to hindbrain and cholinergic preganglionic spinal cord nuclei that via postganglionic catecholaminergic neurons innervate WAT, BAT and muscle (Caverson et al., 1984; Bamshad et al., 1999; Sutton et al., 2014; Doslikova et al., 2019). Activation of CRH neurons or administration of CRH (intracerebroventricular) inhibit food intake and increases thermogenesis (Arase et al., 1988; Krahn et al., 1988, 1990; Holt and York, 1989; Liu et al., 2017). Whereas local administration of TRH into the PVH increases body temperature and blood glucose in rats, activation of TRH neurons which project the ARC increases food intake (Krashes et al., 2014; Z. Zhang et al., 2018). CART overexpression promotes food intake (Douglass et al., 1995; Koylu et al., 2000; Smith et al., 2008); administration of CART in the PVH promotes mRNA expression of uncoupling protein-1 (*UCP1*) which initiates thermogenetic responses in BAT (C.F. Wang et al., 2000). However, PVH ErbB4 deletion did not affect food intake, suggesting that CRH, TRH, and CART neurons of the PVH may not be involved.

OXT/NOS-expressing neurons have been shown to regulate by energy expenditure by controlling the sympathetic outflow (Sutton et al., 2014). Whether ErbB4 is expressed in solely on OXT/NOS+ descending neurons remains unclear. Although ErbB4 is expressed in GABAergic neurons in the cortex, hippocampus, and amygdala (Flames et al., 2004; Woo et al., 2007; Vullhorst et al., 2009; Neddens and Buonanno, 2010; Bean et al., 2014) in other subcortical regions and in the spinal cord, ErbB4+ neurons could be glutamatergic and NRG1 via ErbB4 promotes glutamate release (Bean et al., 2014; H. Wang et al., 2022). It remains unknown whether metabolic regulation by ErbB4 is because of its effect on glutamatergic transmission in the PVH. Therefore, it is likely that ErbB4 within the PVH drives increased energy expenditure through regulation of glutamatergic transmission and neuropeptide release. On the other hand, 63% of ErbB4+ cells in the PVH are not positive for OXT (Fig. 4*E*). It would be interesting to determine whether these OXT- neurons are positive for CRH or CART and whether the metabolic regulation by ErbB4+ neurons involve additional mechanisms.

In sum, we provide evidence that ErbB4 in the PVH plays a role in metabolism, likely by regulating OXT neurons, revealing a novel function of ErbB4. Considering that ErbB4 is a risk gene for both obesity and major depression disorder (Locke et al., 2015; Howard et al., 2019), our study provides insight into pathophysiological mechanisms of depression-associated obesity.

## Synthesis

Reviewing Editor: Tod E Kippin, University of California Berkeley Decisions are customarily a result of the Reviewing Editor and the peer reviewers coming together and discussing their recommendations until a consensus is reached. When revisions are invited, a fact-based synthesis statement explaining their decision and outlining what is needed to prepare a revision will be listed below. The following reviewer(s) agreed to reveal their identity: Bradford Lowell.

Reviewer #1

1. Authors provide reasonably convincing data that loss of ErbB4 in PVH neurons, including oxytocin-expressing PVH neurons, causes obesity that is likely due to a decrease in energy expenditure.

In figures 3 and 5, where the authors report data suggestive of a decrease in energy expenditure, they correct the following parameters, specifically VO2 consumption, VCO2 production and heat generation, for body weight. For many years, it has been noted that correcting such measurements for body weight can lead to spurious conclusions (for example, dividing these parameters by the elevated body weights can lead to incorrect findings of reduced energy expenditure). There are many papers on this topic, including the one cited below.

Müller TD, Klingenspor M, Tschöp MH. Revisiting energy expenditure: how to correct mouse metabolic rate for body mass. Nat Metab. 2021 Sep;3(9):1134-1136. doi: 10.1038/s42255-021-00451-2. Erratum in: Nat Metab. 2021 Oct;3(10):1433. PMID: 34489606.

The authors should reanalyze their data with these issues in mind.

2. In many places throughout the manuscript, the authors seem to suggest that it is the lowering of oxytocin that is responsible for the obesity phenotype, and that perhaps that it the lowering of secreted oxytocin in the blood that is responsible. The authors provide no direct support for this view other than claiming that oxytocin levels are reduced in the knockout mice.

There are two types of oxytocin-expressing neurons in the PVH. Neuroendocrine oxytocin neurons that project to the posterior pituitary and secrete oxytocin into the blood, and hindbrain- and spinal cord-projecting oxytocin neurons that regulate sympathetic outflow. It is the latter that regulate energy expenditure.

The following reference is relevant to this point and should be cited in this paper:

Sutton AK, Pei H, Burnett KH, Myers MG Jr, Rhodes CJ, Olson DP. Control of food intake and energy expenditure by Nos1 neurons of the paraventricular hypothalamus. J Neurosci. 2014 Nov 12;34(46):15306-18. doi: 10.1523/JNEUROSCI.0226-14.2014. PMID: 25392498; PMCID: PMC4228133.

3. Given this, it is much more likely that it is deficiency of ErbB4 in the hindbrain- and spinal cord-projecting neurons that are responsible for the obesity phenotype. Also, as these neurons are glutamatergic, the relative roles of glutamate versus oxytocin release are unknown - hence, in the absence of direct evidence for a role of oxytocin per se, the authors should tone down their claims that the phenotype is caused by a reduction in oxytocin.

Minor Points:

4. The authors should mention the serotype of the AAVs that were employed.

5. Line 223 - “Fig. 2B” should be “Fig. 1D”.

6. Lines 315-322 - The authors investigate whether the PVH neurons expressing Erb4 are GABAergic. I suppose they are doing this because ErbB4-expressing neurons in other parts of the brain are GABAergic. However, two points here: 1) all neurons in the PVH are glutamatergic so looking for GABAergic neurons in the PVH is of unclear value, and 2) one needs to be careful when using GAD to mark GABAergic neurons. Many single neuron sequencing studies have found that GAD can be expressed in neurons that are glutamatergic.

Reviewer #2

The current manuscript presents substantial evidence supporting the hypothesis that ErbB4 expression in the hypothalamic paraventricular nucleus (PVN) is a protein important for the regulation of energy balance. In particular the data suggest that the expression of this tyrosine kinase receptor in oxytocin neurons is important for maintaining a lean phenotype and that selective deletion of this receptor in oxytocin neurons impacts body weight via a reduction of energy expenditure. These data are novel and the experiments are well crafted. The manuscript is very well written and data are presented concisely and effectively. Overall, the data are convincing and I have o only minor suggestions to make.

In figure one, the authors provide an image of a PVN that has been infected with the CRE virus expressing GFP. I, however, know how difficult it is to hit this region exclusively given the size of his structure. I would be interested on seeing if the effects on weight gain and energy expenditure observed in the CRE infused ErbB4 floxed mice varied depending if the location of the infusion only hit one side but not the other, or not at all. This is a comment that I have for all viral infusion experiments.

Also, on Figure 1 there seems to be a red fluorescent signal that is not described in the caption.

Details on the oxytocin ELISA should be added including the intra assay variability and detection limits.

I think the authors should also discuss the possibility of other neuronal populations exist in the PVN co- expressing ErbB4. The authors briefly mention CRH and TRH neurons in the PVH but other populations like CART should also be considered. A good paper to use for this would be that by the Colmers group (https://doi.org/10.1002/cne.24710) showing detailed neuroanatomical distribution of PVN cells projecting to BAT.

Despite these comment, I think this is a nice collection of experiments and hope the authors consider addressing this and other comments from the review panel.
